# About Nystagmus Transformation in a Case of Apogeotropic Lateral Semicircular Canal Benign Paroxysmal Positional Vertigo

**DOI:** 10.1155/2011/687921

**Published:** 2011-07-26

**Authors:** Paolo Vannucchi, Rudi Pecci

**Affiliations:** Department of Surgical Sciences Oto-Neuro-Ophthalmology, Service of Audiology, University of Florence, Viale Morgagni 85 50100 Florence, Italy

## Abstract

There are two forms of lateral semicircular canal benign paroxysmal positional vertigo: geotropic and apogeotropic. When the pathophysiological mechanism of the apogeotropic form is that of canalolithiasis, we can observe a transformation from an apogeotropic nystagmus into a geotropic one. Usually, this phenomenon happens simultaneously on both sides, thus enabling us to observe a right-beating paroxysmal positional nystagmus when the patient lies on the right side and a left-beating paroxysmal positional nystagmus on the left side. We describe a case in which the transformation occurred gradually, so that, after three head rotations from side to side in supine position, there was a right nystagmus beating toward the ground (geotropic) with the patient on the right side and a right nystagmus beating away from the ground (apogeotropic) on the left side. However, after further rotations we observed the nystagmus transformation also on the left side, with a geotropic nystagmus on both sides. The phenomenon of gradual transformation could happen because initially only a part of the debris moved from the anterior to the posterior aspect of the canal during head rotations.

## 1. Introduction

Benign Paroxysmal Positional Vertigo (BPPV) is the most frequently found type of vertigo in clinical practice. The posterior semicircular canal (PSC) is more frequently involved, the lateral semicircular canal (LSC) less frequently so, and the anterior semicircular canal (ASC) only rarely [1–3]. The LSC-BPPV is described in two forms [4, 5]: (a) the geotropic form, in which the direction of the fast phase of the nystagmus is right when the patient lies on the right side and left when the patient lies on the left side; (b) the apogeotropic form, with a right nystagmus when the patient lies on the left side and a left nystagmus when the patient lies on the right side. When the nystagmus is geotropic, the debris is located in the posterior aspect of the canal, and the pathophysiological mechanism is a canalolithiasis [5]. When the nystagmus is apogeotropic, the mechanism can be a canalolithiasis, with the debris within the anterior aspect of the canal, or a cupulolithiasis, with the debris attached to the cupula (on its canal or utricular wall) [6]. In some cases, the apogeotropic form changes into the geotropic one. In this case the debris moves from the anterior aspect into the posterior aspect of the canal. Thus, in a patient who initially shows a left beating nystagmus when lies on the right side and a right beating nystagmus when lies on the left side,we later observe a right beating nystagmus when the patient lies on the right side and a left beating nystagmus when he/she lies on the left side. We have noted this transformation always at the same time on both sides; only in the patient described in this paper did we note the transformation earlier on one side and, after more head rotations in supine position, also on the other side. We will now describe the case and make a few conjectures as regards the pathophysiological mechanisms involved.

## 2. Material and Method

After collecting a detailed history the patient underwent a microscope otologic inspection and an audiometric and impedance testing; then we looked for spontaneous, gaze-evoked, rebound, and positional nystagmus, both with and without fixation (in the latter with infrared video cameras and nystagmus recording). We performed the Head Shaking Test (HST), the Head Impulse Test, and the caloric test (according to the Fitzgerald-Hallpike method) in order to study the canal paresis and directional preponderance. Lastly, we tested for the cervical Vestibular Evoked Myogenic Potentials (cVEMPs) and the Subjective Visual Vertical (SVV). 

After the diagnosis, the therapy was a forced prolonged position in which the patient was asked to lie for eight–ten hours on the healthy side [7]. Informed consent was obtained from the patient.

## 3. Case Report

The patient was a 73-year-old male, who suffered from right Menière's disease for the past 10 years, with normal Computerized Tomography scan and Magnetic Resonance Imaging. In both 1996 and 2004, two infiltrations of transtympanic gentamicin were performed and a good control of the disease was obtained, so that the patient did not complain any more of spontaneous vertigo lasting many hours, as typical of Menière's disease. 

However, in December 2006 the patient reported positional and short-lasting vertigo triggered by moving his head from side to side in supine position. The audiogram showed a flat-type hearing loss with PTA to 80 dB on the right and a presbyacusis with pure tone threshold, from 500 to 4000 Hz, of 15,15, 30, 50, and 65 dB on the left. There was a caloric weakness in the right ear, with a canal paresis of about 50%, but both the Head Thrust Test and the HST were negative. The cVEMPs were normal on the left and absent on the right; the SVV was normal. 

We studied the nystagmus with and without fixation using infrared video cameras. When the patient was moved from a sitting position to a supine one, a small, horizontal, left-beating nystagmus, lasting about two minutes, was observed. When the patient's head was rotated by 90° to the right, a more intense left-beating nystagmus (apogeotropic) appeared. When he moved his head by 180° to the left, a more intense right-beating (still apogeotropic) nystagmus was observed. In the following two rotations from side to side, we again observed an apogeotropic nystagmus on both sides albeit more intense with the head in the right-ear-down position. 

In rotating the patient for the third time onto his right side, we noted a right-beating geotropic nystagmus thus obtaining a transformation from apogeotropic nystagmus into a geotropic one; but, in rotating the patient onto his left side, a right apogeotropic nystagmus was observed. The latter nystagmus was less intense than the previous one observed on the same (left) side. When we repeated the head rotations from side to side, we again observed a right beating geotropic nystagmus on the right side, and a right beating apogeotropic nystagmus on the left side, that became progressively less intense at each positioning. When moving the patient for the fifth time on his right side there was still a right nystagmus but rotating the patient on his left side, after a pause of three seconds, we observed a very violent left-beating geotropic nystagmus that was associated with intense vertigo and autonomic symptomatology. Now, because of a more intense geotropic nystagmus on the left side, we can hypothesize a left LSC-BPPV, then on the opposite ear to that with Menière's disease.

Therefore, the transformation from the apogeotropic to the geotropic form happened gradually only after a lot of side-to-side head rotations and needed as long as forty minutes to occur.

The following night the patient stayed on his healthy (right) side for eight–ten hours (Forced Prolonged Position) [7], and on the next check-up LSC-BPPV was resolved because the nystagmus and symptomatology disappeared.

## 4. Discussion

There are two types of LSC-BPPV: geotropic and apogeotropic [4–7]. Geotropic LSC-BPPV is more frequent, and is caused by a canalolithiasis [8]. Apogeotropic LSC-BPPV is less frequent and may be related to canalolithiasis, with debris in the anterior aspect of the lateral canal, or to cupulolithiasis [9] with debris attached to the cupula on the vestibular or canalar side. In both cases of apogeotropic LSC-BPPV (canalolithiasis or cupulolithiasis), an ampullofugal current happens when the patient lies on the affected side; when the patient lies on the healthy side, an ampullopetal current occurs. 

How does one explain the transformation from apogeotropic to geotropic nystagmus in LSC-BPPV? In the canalolithiasis, if the debris moves from the anterior aspect to the posterior aspect of the canal, the apogeotropic form could become geotropic. In cupulolithiasis, transformation could be more difficult: for example, if the debris is on the canalar side of the cupula, it could become detached and, thus free to move in the canal, could transform the cupulolithiasis into canalolithiasis. If the debris is on the vestibular side of the ampullar receptor, the transformation could happen only if the debris detached from the cupula enters the vestibule and then goes into the posterior aspect of the LSC (it is very difficult that this phenomenon occurs: it is more probable that there is a resolution of the vertigo without any transformation). When the transformation happens, however, the apogeotropic nystagmus becomes geotropic irrespective of patient's side positions (i.e. right or left side position).

Instead, in this particular case study, the transformation happened gradually, first on one side and then on the other.

**Figure 1:**
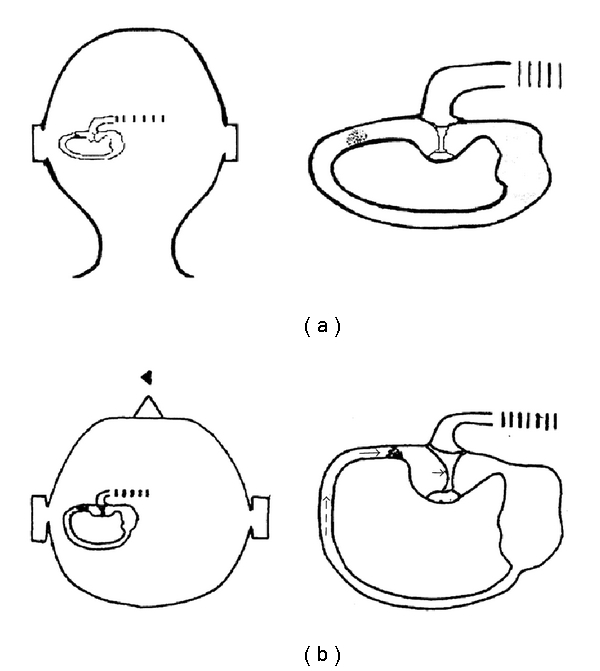
(a) Patient with a left LSC-BPPV of the apogeotropic form in sitting position (backward view): the debris is localized in the anterior arm of the canal and is free floating. (b) When the patient moves from the sitting to the supine position, the endolymphatic flow (dashed arrow) causes the debris to move towards the ampulla (thin arrow). Here, acting as a plunger, debris determines an ampullopetal deflection of the cupula (thick arrow), thus triggering a left-beating nystagmus (head arrow).

We can conjecture that, in the beginning, the debris was inside the anterior aspect of the left lateral canal because of the left beating nystagmus that resulted from moving the patient from a seated to a supine position [10] (Figure 1), and because, by rotating the head by 180° from side to side, the nystagmus on the right side was stronger than that on the left side. Moreover, at the end of our study of this patient, we were to learn that the lateral canal involved really was the one on the left.

**Figure 2:**
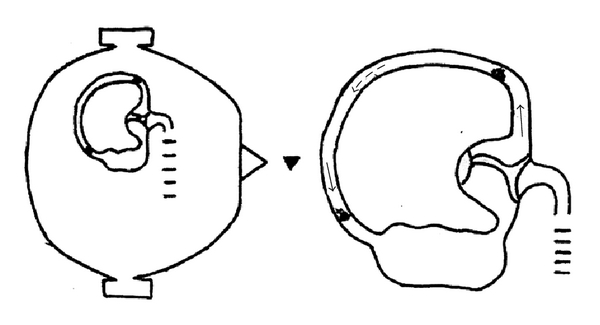
When the patient in supine position rolls his/her head by 180° from the left to the right side for the third time, pushed by the endolymphatic flow (dashed arrow), some of the debris moves into the posterior arm of the canal, while another part remains in the anterior arm, both moving in an ampullofugal way (thin arrows), thus triggering a right beating geotropic nystagmus (head arrow).

**Figure 3:**
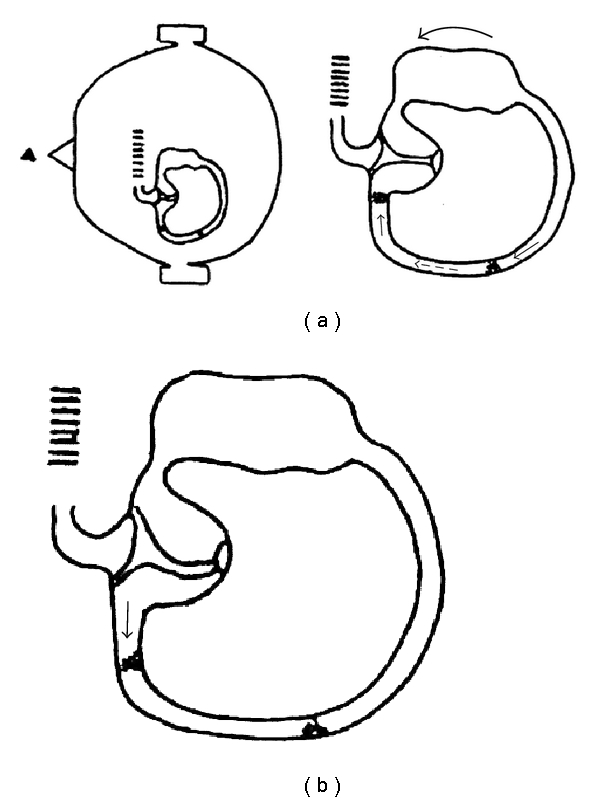
(a) When the patient in supine position rolls his/her head by 180° from the right side to the left side (outer arrow), the endolymphatic flow (dashed arrow) pushes the debris localized in the anterior arm of the canal towards the cupula and those in the posterior arm towards the lowermost part of the canal (thin arrows). (b) Therefore, when the head is still in this position, the debris in the anterior arm of the canal moves away from the ampulla due to gravity (thin arrow), provoking an ampullofugal flow and a right beating apogeotropic nystagmus (head arrow), while the debris in the posterior arm does not provoke any flow.

**Figure 4:**
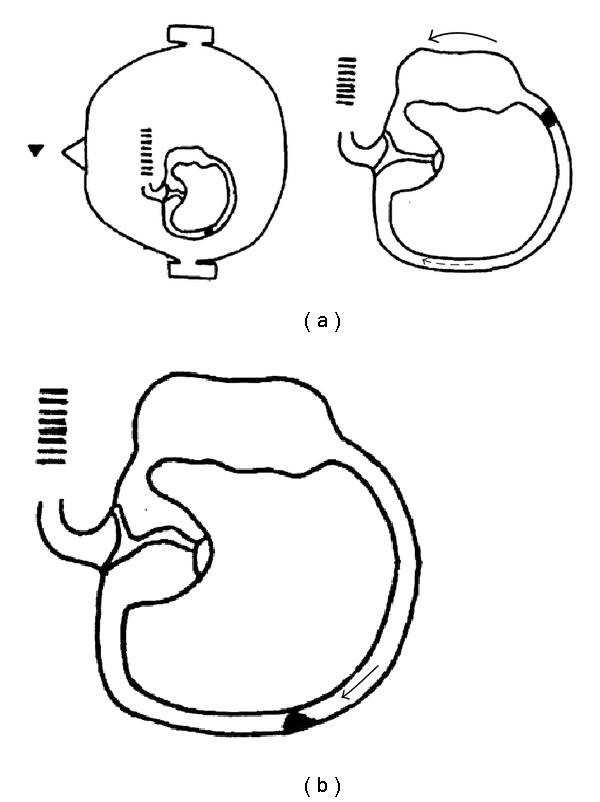
(a) In the end, when the patient in supine position moves his/her head from the right side to the left side (outer arrow), the endolymphatic flow (dashed arrow) is not able to move the debris in the posterior part of the canal towards the ampulla due to their weight. (b) It is only when the head is still that the debris suddenly falls, due to gravity, towards the cupula (thin arrow), provoking a violent left beating geotropic nystagmus (head arrow).

In rotating the patient's head in supine position from the left side to the right side for the third time, we observed a right beating geotropic nystagmus. This could be explained by the movement of the debris from the anterior aspect to the posterior aspect of the canal. But by moving the head to the left side, we did not observe a left beating geotropic nystagmus, but again a right beating nystagmus that was apogeotropic lying the patient on the left side. How do we explain this phenomenon? It is possible that during the rotation towards the right side only a part of the debris moved from the anterior to the posterior aspect of the canal, while another part remained in the anterior aspect (Figure 2). By moving the head towards the left side, the current pushed the debris localized in the anterior aspect towards the cupula and that in the posterior aspect towards the central part of the canal. Thus, with the patient remaining in this position, the debris in the anterior aspect fell towards the vestibule, provoking an ampullofugal current and an apogeotropic nystagmus, while the debris in the posterior aspect did not provoke any current (Figure 3). By moving the head again towards the right side, other debris moved from the anterior aspect to the posterior aspect, causing an ampullofugal current with a right beating geotropic nystagmus. In the end, all the debris was in the posterior part of the canal, so that, by moving the head towards the left side, the current was not able to move this heavier than endolymph material towards the ampulla. After a brief latency, the debris fell towards the cupula only when the head was motionless, due to gravity, provoking a very violent left geotropic nystagmus (Figure 4).

## 5. Conclusion

Some cases of apogeotropic LSC-BPPV are transformed into geotropic form, and others resolve directly. Moreover, usually the transformation is observed on both sides when the patient in supine position rotates his/her head from side to side. In this case study, the transformation occurred gradually: first on the right side we observed a right beating geotropic nystagmus but on the left side we again observed a right beating apogeotropic nystagmus; then, after other head rotations, we observed a geotropic nystagmus on both sides (with a left beating geotropic nystagmus even on the left side). In the end, the clinical picture was typical of geotropic form LSC-BPPV.

What can this case teach us? To explain the nystagmus that we observe during the examination of the vertiginous patients, it is necessary to consider the position of the patient, the plane and the direction of the head's movements, the current provoked by the movement, and the action of gravity on the debris when the head is still. We can try to explain the nystagmus observed only if we keep in mind all of these factors.
